# Oculomotor indicators of cognitive performance are modulated by neurodegeneration

**DOI:** 10.3389/fneur.2025.1649745

**Published:** 2025-11-10

**Authors:** Patrice Voss, Nils A. Koch, Maryse E. Thomas, Paul S. Giacomini, Etienne De Villers-Sidani

**Affiliations:** 1 Montreal Neurological Institute, McGill University, Montreal, QC, Canada; 2 Innodem Neurosciences, Montreal, QC, Canada; 3 Integrated Program in Neuroscience, McGill University, Montreal, QC, Canada

**Keywords:** cognition, eye-tracking, oculomotor dynamics, saccades, Parkinson’s disease, predictive model

## Abstract

In this study, the extent to which eye movements can be used to estimate cognitive ability in neurologically intact individuals was evaluated in the absence of clear underlying neurodegenerative processes. In contrast to previous studies of Parkinson’s Disease (PD) and multiple sclerosis that demonstrated a strong link between oculomotor parameters and clinical measures of cognition, this relationship is unaffected by disease in healthy participants, enabling a more direct assessment of the connection between eye movements and cognition. Accordingly, a modest portion (≤28%) of the observed variance in cognitive test scores could be explained by oculomotor parameters in 204 participants aged 18–79 with no differences between males and females observed. The relationship between oculomotor parameters and cognitive measures was further compared between neurologically intact individuals and a separate sample of 65 individuals with PD. Oculomotor parameters showed stronger correlations with cognitive measures in PD patients, likely contributing to the greater explanatory power of oculomotor-based models in this population. Finally, given that many oculomotor parameters are affected by age, the ability to estimate an individual’s age without confounding neurodegeneration was assessed. As 33% of the variance in participants’ age could be explained by oculomotor parameters, age may be estimated from oculomotor parameters, providing insight into the aging brain. Collectively, these findings highlight the connection between oculomotor function and clinical measures of cognition in the absence of neurodegeneration and indicate that these relationships are likely mediated by the functional integrity of brain networks involved in both motor control and cognitive processing.

## Introduction

Although knowledge about our ability to infer brain function through analysis of eye movements has been around for a few centuries (1), the advent of modern eye-tracking technology has resulted in a growing interest in probing brain circuitry integrity via the detailed study of eye movements. Much of the recent work has attempted to develop eye-movement and gaze-based markers of neurodegenerative processes, such as Alzheimer’s Disease (AD), Parkinson’s Disease (PD), and Multiple Sclerosis (MS). For instance, AD is associated with difficulty in inhibiting incorrect responses during an anti-saccade task and with a reduction in directional error corrections (2, 3), and while findings have not been consistent across all studies, some have also reported an increased rate of saccadic intrusions during fixation (4, 5). Hallmark features of PD also include hypometric saccades toward targets, which also often follow a multistep sequence (6, 7). Additionally, internuclear ophthalmoplegia (INO), a slowing of the adducting eye during horizontal saccades, is a well-documented oculomotor feature of MS (72), with reported prevalence estimates ranging from 15 to 52%, depending on the assessment method used [see Hof et al. (8) for a more comprehensive overview].

Beyond identifying markers of disease, a growing body of research suggests that it may be possible to infer disease severity and track disease progression via the analysis of eye movements. Several studies have indicated that individual oculomotor parameters (e.g., saccadic latency or anti-saccade error rate) correlate strongly with clinical scale scores [e.g., the Expanded Disability Status Scale (EDSS) for MS or the Movement Disorder Society Unified Parkinson’s Disease Rating Scale (MDS-UPDRS III) (9–12)]. Although cognition has often been investigated via eye-tracking paradigms of free-viewing conditions, where coarse eye movement and gaze parameters are interpreted to reflect certain cognitive processes such as memory and attention (13–15), the study of cognitive function via detailed oculomotor analysis is relatively novel. Indeed, recent evidence suggests that cognitive ability can be inferred via the analysis of spatiotemporal parameters measured with infrared eye-tracking devices and standard paradigms of oculomotor function (e.g., pro-saccades, fixation stability, anti-saccades, and smooth pursuit tasks). For instance, several oculomotor metrics measured in individuals with PD have been shown to significantly correlate with measures of general cognition such as the Mini-Mental Status Exam (MMSE) (16, 17) or the Montreal Cognitive Assessment (MoCA) (18, 19), or with measures of processing such as the Symbol Digit Modalities Test (SDMT) (20, 21) and Paced Auditory Serial Addition Test (PASAT) (22, 23) in MS patients.

Using a novel mobile eye-tracking software that functions using the standard camera of an iPad Pro (Eye-Tracking Neurological Assessment (ETNA™); Innodem Neurosciences), our group recently replicated many of the aforementioned findings in several clinical populations. In particular, several oculomotor parameters, when jointly considered, could account for a large proportion of the variance in cognitive test scores. For instance, a combination of oculomotor parameters and machine learning regression models was able to explain between 43 and 72% of the variance on cognitive test scores in PD patients (24), and explain between 48 and 73% of the variance on cognitive test scores in MS patients (25).

However, to our knowledge, most of the evidence described above linking cognitive ability to oculomotor parameters was obtained in clinical populations, particularly those with neurological disorders. Notably, disease-related factors may simultaneously affect both eye movements and cognitive function, making it difficult to determine whether observed relationships reflect direct associations or shared consequences of the underlying condition. Studying neurologically intact individuals offers a unique opportunity to disentangle these effects by providing a clearer baseline from which to understand the specific contribution of cognitive function to oculomotor measures. As previous studies have demonstrated that oculomotor function is a sensitive marker of neural integrity in various neurological conditions (26–28), the current study aims to extend these findings by examining this relationship in healthy individuals.

As such, the primary objective of the present study was to determine to what extent cognitive ability could be estimated in a large sample of neurologically intact individuals via oculomotor parameters measured with standard eye-tracking tasks (i.e., pro-saccades, anti-saccades, smooth pursuit, fixation, optokinetic nystagmus). To evaluate cognitive ability, four of the cognitive domains outlined in the Movement Disorder Society Task Force Guidelines (29) were measured for comparison with previous findings in participants with PD (24) using the following tests: MoCA (global cognitive), Trail Making Test (TMT A/B) (attention and working memory), Controlled Oral Word Association Test (COWAT) of verbal fluency (executive function), Hopkins Verbal Learning Test (HVLT; memory). The Symbol Digit Modalities Test (SDMT) was additionally included to assess cognitive processing speed and the Beck Anxiety Inventory (BAI) to assess participant anxiety levels as part of the assessment.

Correlations between clinical test scores, participant age, and all individual oculomotor parameters were investigated. Subsequently a partial least squares (PLS) regression approach was used to determine the extent of clinical score variance that could be explained using the eye movement parameters. A secondary objective was to determine to what extent an individual’s age could be estimated in the absence of confounding neurodegeneration, given that many oculomotor parameters are known to be impacted by age (30–32). How well this age estimate can also explain clinical score variance was then examined. Finally, the relationship between oculomotor inputs and cognitive scores and the performance of the PLS regression models of neurologically intact participants was compared with those obtained from a sample of PD participants. In light of a growing body of evidence highlighting critical sex differences across numerous domains of neuroscience (33), the data collected in this study were disaggregated by sex wherever possible to explore potential sex-related effects—an approach that remains underutilized in much of neurological research. Although the study was not explicitly designed to investigate sex differences, the relatively large sample size would enhance confidence in any observed effects and could support the generation of testable hypotheses for future targeted investigations.

## Methods

### Subject populations

#### Healthy group

204 cognitively and neurologically intact (self-reported) individuals took part in this study (Healthy Controls (HC): age 40.2 ± 15.0, range 18–79, 120/84 males/females). All individuals provided informed and written consent and were recruited from the general public. The study procedures outlined in this paper were approved by and performed in accordance with the guidelines of the Veritas and the McGill University Health Centre research ethics boards.

#### PD group

For model comparison purposes, data collected from 65 Parkinson’s Disease (PD) patients with mild-to-moderate idiopathic PD used in two previous publications (24, 34) were also used in the present study to compare the oculomotor-based predictive models between the PD patients and the neurologically intact individuals. All PD patients (age 64.1 ± 8.4, range 45–89, 43/22 males/females) were diagnosed by a movement disorder specialist in the province of Quebec according to the MDS criteria (35) and were enrolled as part of the Quebec Parkinson Network (QPN; https://rpq-qpn.ca/) initiative (36). Data was collected in a sample of 65 consecutively recruited patients. Additional patient details can be found in Koch et al. (24).

### Oculomotor assessment and experimental setup

All eye-tracking tests were performed using the ETNA™ software installed on a 12.9-inch iPad Pro tablet. The software enables the simultaneous presentation of visual stimuli on-screen and video recordings of the eyes using the embedded front-facing camera at 60 frames per second. Gaze-tracking is performed in visible light with a deep neural network that uses four inputs to produce a general gaze model: an image of each of the user’s eyes, an image of the user’s face, and the Euler angles of the head as head pose information. Apple’s ARKit was used to detect facial landmarks.

Participants were seated throughout the experiment. The iPad was positioned vertically using an adjustable tablet stand, placed approximately 45 cm from the participant, such that the center of the screen aligned with eye level. While no physical head restraints (e.g., chin rests) were used, participants were instructed to minimize head movement during the tasks. The ETNA™ software includes built-in safeguards to monitor head orientation and eye-to-screen distance in real time. If the participant’s head position deviated beyond acceptable limits, an on-screen prompt provided clear visual instructions for realignment (e.g., “Please move your head slightly forward” or “Please tilt your head slightly to the left”), thereby ensuring consistent data quality throughout the assessment.

All participants performed five oculomotor tasks in the following order: a fixation task, a pro-saccade task, an anti-saccade task, a smooth pursuit task, and an optokinetic nystagmus (OKN) task. Task details and parameters are outlined in the Supplementary materials. All participants performed a brief calibration step whereby they tracked a slow-moving target on-screen using the ETNA™ software, before undertaking the visual tasks. The calibration procedure itself trains an additional model, which is then incorporated into the general gaze model to produce the final individualized gaze-tracking model. The ETNA™ software’s gaze-tracking algorithms have an estimated average (over the entire screen) accuracy of 0.47 degrees (mean offset between the actual gaze position and the recorded gaze position) and precision of 0.33 degrees (as calculated via Root Mean Square (RMS) of the sampled points); an estimate of reliability of the gaze point estimate from one sample to the next, which are comparable values to those of research-grade infrared eye tracking devices. For a detailed description of how oculomotor parameters were extracted from the gaze signal, see Supplementary materials.

### Clinical and cognitive assessments

Cognitive assessments included the following: the Montreal Cognitive Assessment (MoCA) (37), the Trail Making Test (TMT A/B) (38), the Hopkins Verbal Learning Test (HVLT) (39), and the CFL version of Controlled Oral Word Association Test (COWAT) (40). These tests were selected as they had been administered to most PD patients enrolled in the Quebec Parkinson Network initiative and they evaluated four of the cognitive domains outlined in the Movement Disorder Society Task Force Guidelines (29). For healthy participants, we included an additional measure of cognitive processing with the Symbol Digit Modalities Test (SDMT) – (41) and of state anxiety with the Beck Anxiety Inventory [BAI – Beck et al. (42)]. The number of HC and PD participants who completed each test is as follows: MoCA (203 HC, 36 PD), TMTA (203 HC, 50 PD), TMTB (203 HC, 49 PD), COWAT (203 HC, 48 PD), HVLT (200 HC, 50 PD), SDMT (198 HC), BAI (203 HC).

### Clinical outcome measures

Given the number of tests used and the multiple possible outcome measures for each, we selected, *a priori*, one outcome measure per test for use in subsequent analyses. MoCA: the total score (out of 30) was used as the outcome measure. SDMT: the total number of correct symbol-digit pairings within a 90 s time limit (out of 110). TMT – Part A (TMTA): time in seconds to connect numbered circles in ascending order. TMT – Part B (TMTB): time in seconds to connect the numbers and letter sequence alternately. HVLT: total recall score, which is the sum of the correctly recalled words across the three learning trials. COWAT: total count of valid words produced during one minute per letter, using letters C-F-L. BAI: total score (out of 63).

### Data analyses

#### Correlation analyses

For all correlations between eye movement parameters and the demographic or clinical outcome measures of interest (Age, MoCA, TMTA, TMTB, HVLT, COWAT, and BAI), the Spearman’s rho correlation coefficient was calculated. This rank-based approach was selected because it does not assume linearity or normally distributed variables, and it is more robust to outliers than Pearson’s correlation, which was important given the skewed distributions observed for several clinical measures (e.g., Trail Making Test times, BAI scores). We note that Spearman’s rho assumes independent and identically distributed (iid) pairs and can be influenced by ties in rank assignment. Each participant contributed a single set of measures, satisfying independence, and ties were rare for continuous oculomotor parameters. For cognitive scores with more frequent ties (e.g., MoCA, SDMT), average ranks were assigned by the software, an approach demonstrated to introduce minimal bias in large samples (43, 44). We therefore judged Spearman’s rho to be the most appropriate balance between robustness and sensitivity for the present dataset. To adjust for the false discovery rate, corrected *p*-values were computed using the Benjamini–Hochberg procedure evaluated at an alpha level of 0.05.

#### Partial least squares regression analysis

Partial least squares (PLS) regression was used to examine the relationship between oculomotor parameters and each clinical score (Age, TMTA, TMTB, COWAT, SDMT, MoCA, HVLT, and BAI) while accounting for multicollinearity between oculomotor parameters. In order to include subjects with structurally missing values in the oculomotor parameters and maximize sample size input into the PLS models, probabilistic principal component analysis (PPCA) imputation (45, 46) was used to estimate the missing values. A multi-step feature selection procedure was used for each model. The first step consisted of a correlation-based feature selection to determine the 20 parameters (out of a total of 199) most correlated with the clinical score. Subsequently, an exhaustive feature selection procedure was used to select the parameter set of the final model, which involved sampling all possible combinations of those 20 oculomotor parameters (set sizes from 1 to 20) and subsequent model fitting. For each PLS regression model, the number of latent variables maximizing the covariance between the independent and dependent variables was selected by minimizing the Bayesian information criterion (47, 48) using a 10-fold cross-validation approach. The coefficient of determination (*R*^2^) was used to assess multiple regression performance (both adjusted and non-adjusted values).

#### Permutation tests

Permutation tests were used to determine if there was a statistically significant difference between Spearman’s rho correlations or adjusted *R*^2^ between groups (i.e., male vs. female or HC vs. PD). For both measures, the difference in Spearman’s rho or adjusted *R*^2^ between groups was first computed. A null distribution was then generated by shuffling the group labels *N* = 1,000 times and recalculating the difference on each replicate. A two-tailed *p*-value evaluated at an alpha level of 0.05 was obtained by calculating the proportion of permuted differences more extreme than the observed value.

## Results

### Relationship between clinical measures and age

**Figure 1:**
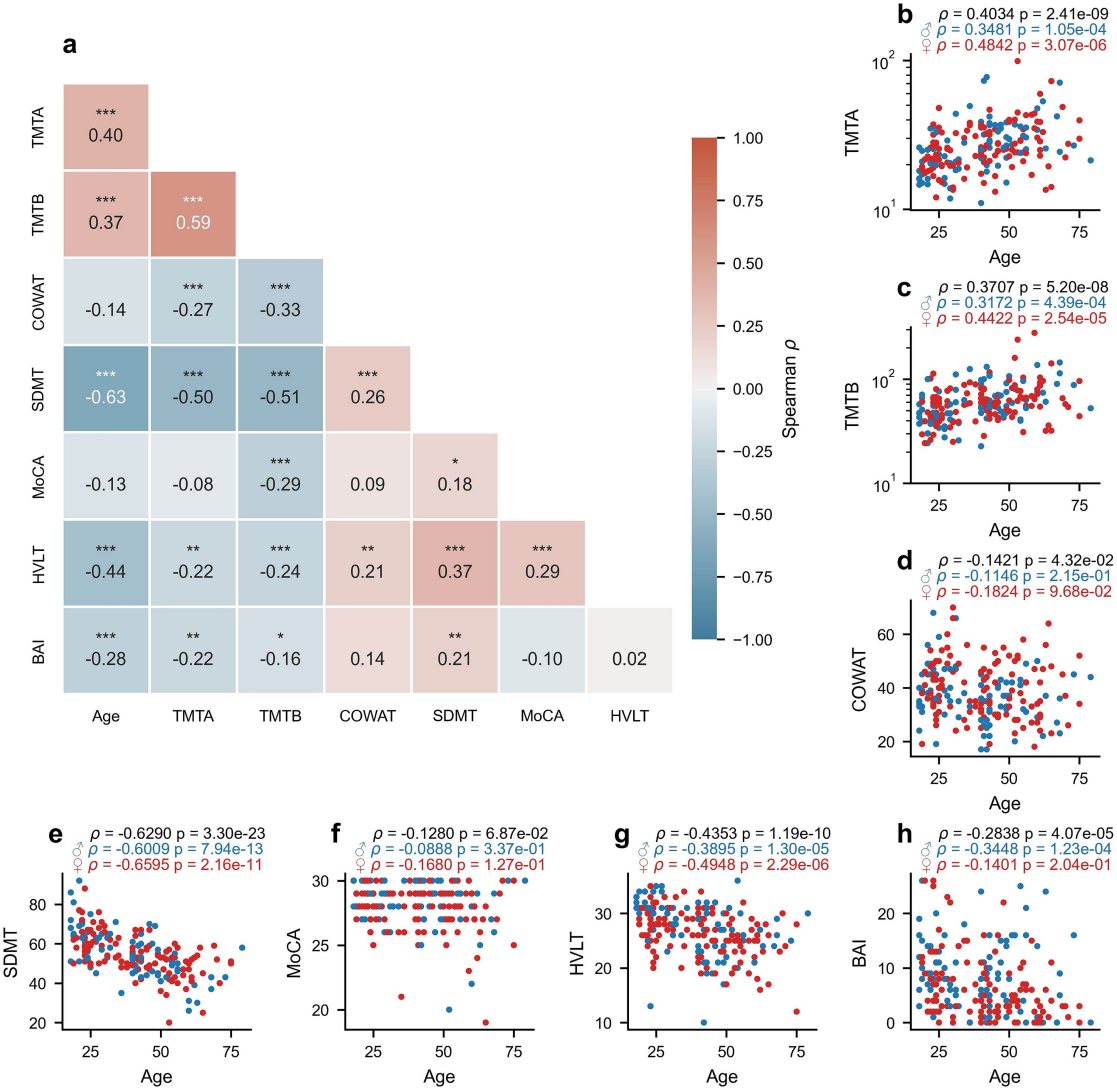
Relationship between age and clinical measures. Spearman’s rho correlation values between each clinical score for healthy individuals **(a)**. Scatterplots highlighting the relationship between age and TMTA **(b)**, TMTB **(c)**, COWAT **(d)**, SDMT **(e)**, MoCA **(f)**, HVLT **(g)**, and BAI **(h)**. Blue dots represent male participants and red dots represent female participants. Spearman’s rho and corresponding *p*-values are reported for the entire healthy participant dataset (black), for females only (red) and males only (blue) above panels b-h. * *p* < 0.05, ** *p* ≤ 0.01, *** *p* ≤ 0.001 (corrected *p*-values).

The correlation coefficients (Spearman’s rho) between all clinical and demographic variables (Age, MoCA, TMTA, TMTB, HVLT, COWAT, BAI) of the healthy group are displayed in Figure 1a (and further documented in Supplementary Table 1). Related test scores unsurprisingly correlated more highly with one another (such as the TMTA and TMTB), whereas scores of general cognition (MoCA) or anxiety levels (BAI) correlated more weakly with the other clinical scores measuring specific cognitive abilities. The relationships between participant age and cognitive/clinical test scores are further depicted for TMTA (Figure 1b, Spearman’s rho = 0.4034, *p* = 2.41*10^−9^), TMTB (Figure 1c, Spearman’s rho = 0.3707, *p* = 5.20*10^−8^), COWAT (Figure 1d, Spearman’s rho = −0.1421, *p* = 0.0432), SDMT (Figure 1e, Spearman’s rho = −0.6290, *p* = 3.30*10^−23^), MoCA (Figure 1f, Spearman’s rho = −0.1280, *p* = 0.0687), HVLT (Figure 1g, Spearman’s rho = −0.4353, *p* = 1.19*10^−10^), and BAI (Figure 1h, Spearman’s rho = −0.2838, *p* = 4.07*10^−5^), which are disaggregated by participant sex (Figures 1b–h). No significant differences were found between the correlation coefficients of males and females for TMTA (Figure 1b, *p* = 0.2680), TMTB (Figure 1c; *p* = 0.3610), COWAT (Figure 1d, *p* = 0.6225), SDMT (Figure 1e, *p* = 0.5240), MoCA (Figure 1f, *p* = 0.5950), HVLT (Figure 1g, *p* = 0.3650), or BAI (Figure 1b, *p* = 0.130) with age, computed via permutation tests (*N* = 1,000 replicates).

### Correlations between eye-tracking parameters and clinical outcome measures

**Figure 2:**
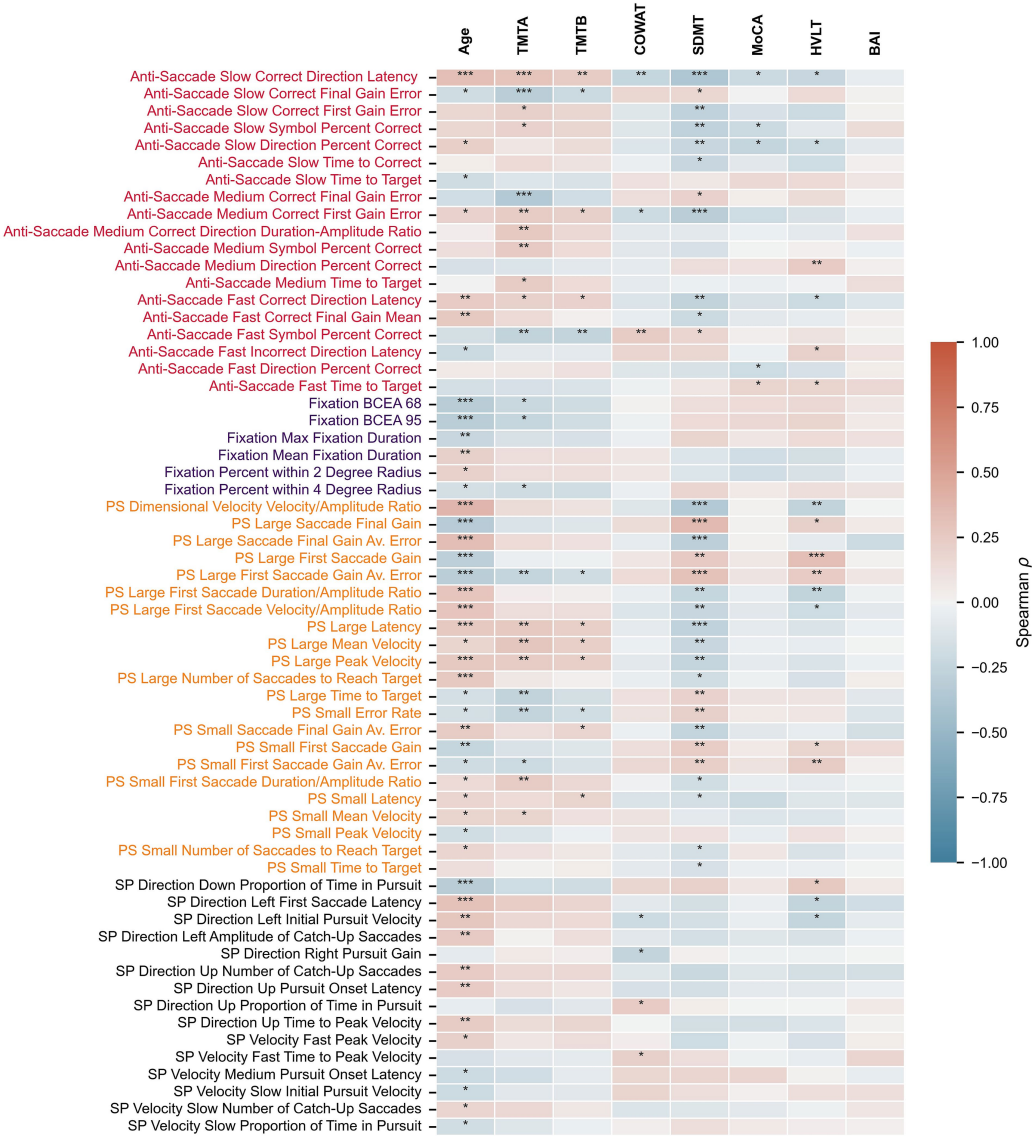
Relationship between oculomotor parameters and clinical measures. Heatmap depicting Spearman’s rho correlation values between eye-tracking parameters and age or clinical scores. Only oculomotor parameters with at least one significant correlation after correcting for multiple comparisons are shown. PS, pro-saccade; SP, smooth pursuit. * *p* < 0.05, ** *p* ≤ 0.01, *** *p* ≤ 0.001 (corrected *p*-values).

**Figure 3:**
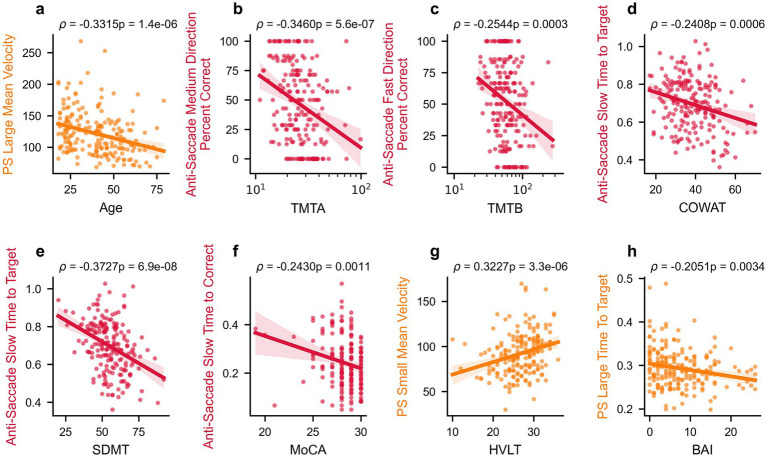
Select relationships between oculomotor parameters and clinical measures. Correlations between select oculomotor parameters and age or clinical scores in healthy participants: Age **(a)**, TMTA **(b)**, TMTB **(c)**, COWAT **(d)**, SDMT **(e)**, MoCA **(f)**, HVLT **(g)**, and BAI **(h)**. Spearman’s rho correlation values and *p*-values are reported above each panel, and linear regression lines with 95% confidence intervals are shown. PS, pro-saccade.

Spearman correlations between the extracted oculomotor parameters and clinical/demographic variables (Age, TMTA, TMTB, BAI, MoCA, COWAT, SDMT, HVLT) are detailed in Supplementary Table 2, with oculomotor parameters having at least one significant correlation shown in Figure 2. Participant age correlates the most strongly with various sets of oculomotor parameters, primarily those from the pro-saccade task. SDMT, TMTA TMTB, and HVLT also had multiple significant correlations with various parameters, particularly from the anti-saccade and pro-saccade tasks. In contrast, MoCA, COWAT, and BAI are significantly correlated with fewer oculomotor parameters. After correction for multiple comparisons, 47 oculomotor parameters were significantly correlated with age, 31 with SDMT, 23 with TMTA, 17 with HVLT, 12 with TMTA, 7 with COWAT, 5 with MoCA, and none with BAI. Select representative significant correlations between clinical outcome measures and oculomotor parameters are depicted in Figure 3.

### PLS regression analyses

**Figure 4:**
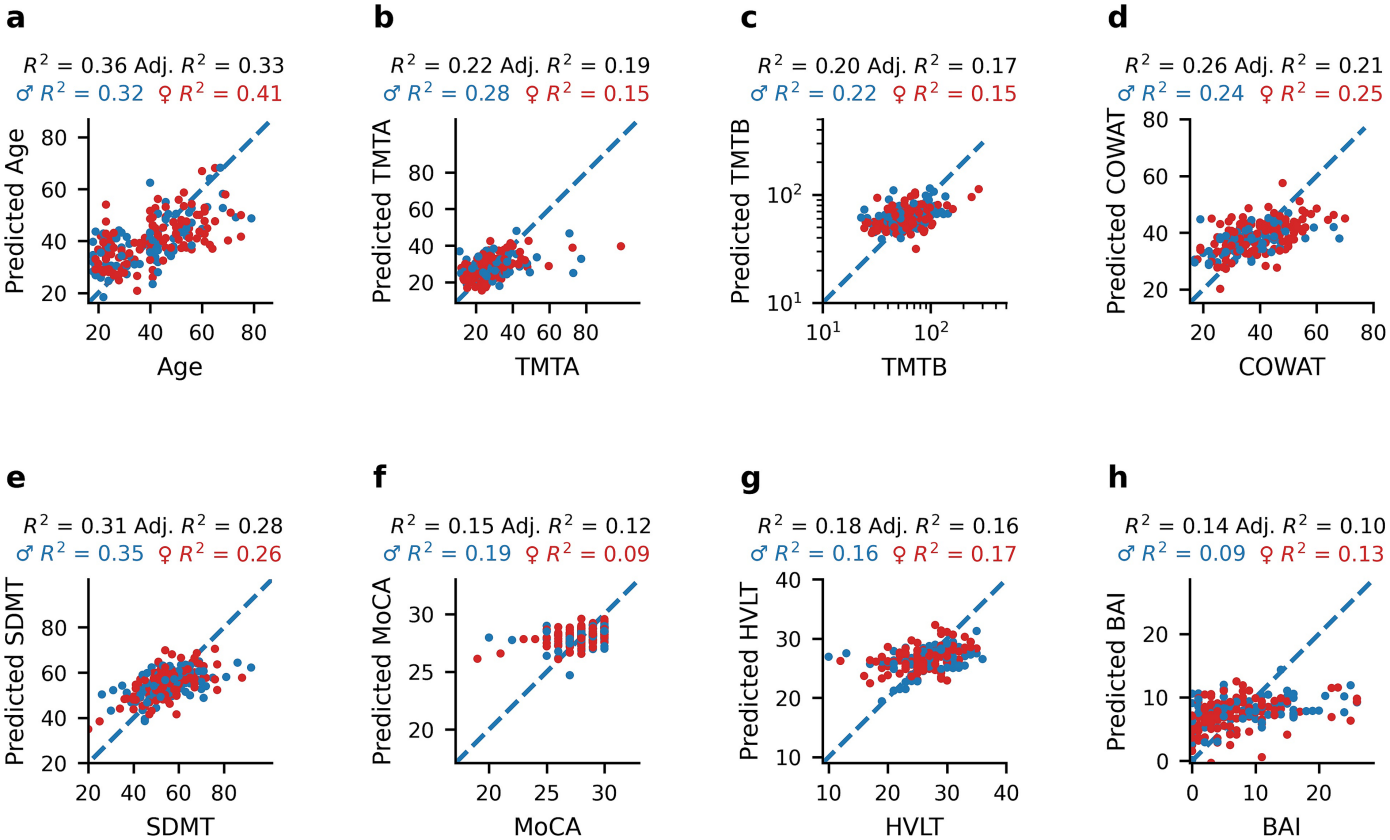
Predictions of age and clinical measures from oculomotor parameters using PLS regression models. Scatterplots of healthy participants’ age and clinical scores compared to the corresponding predicted value obtained by PLS regression analysis using oculomotor parameters as predictors: Age **(a)**, TMTA **(b)**, TMTB **(c)**, COWAT **(d)**, SDMT **(e)**, MoCA **(f)**, HVLT **(g)**, and BAI **(h)**. Blue dots represent male participants and red dots represent female participants. *R*^2^ and adjusted *R*^2^ values are reported for the entire healthy dataset (black), as well as *R*^2^ values for females only (red), and males only (blue) above each panel.

We performed partial least squares (PLS) regression analyses to develop models that best explained each demographic and clinical outcome measure based solely on oculomotor parameters. PLS regressions for each measure are presented in Figure 4 and show that all models explain between 10 and 33% of the variance of the cognitive-, age- and anxiety-related outcome measures. The best variance explanatory model was for age (Figure 4a; *R*^2^ = 0.36, Adjusted *R*^2^ = 0.33), followed by SDMT (Figure 4e; *R*^2^ = 0.31, Adjusted *R*^2^ = 0.28), COWAT (Figure 4d; *R*^2^ = 0.26, Adjusted *R*^2^ = 0.21), TMTA (Figure 4b; *R*^2^ = 0.22, Adjusted *R*^2^ = 0.19), TMTB (Figure 4c; *R*^2^ = 0.20, Adjusted *R*^2^ = 0.17), and HVLT (Figure 4g; *R*^2^ = 0.18, Adjusted *R*^2^ = 0.16). The MoCA (Figure 4f; *R*^2^ = 0.15, Adjusted *R*^2^ = 0.12) and BAI models (Figure 4h; *R*^2^ = 0.14, Adjusted *R*^2^ = 0.10) explained the least variance. Although the PLS regression *R*^2^ values sometimes differed between males and females, no statistically significant differences were observed for age (Figure 4a, *p* = 0.4702), TMTA (Figure 4b, *p* = 0.1824), TMTB (Figure 4c, *p* = 0.5816), COWAT (Figure 4d, *p* = 0.8258), SDMT (Figure 4e, *p* = 0.3578), MoCA (Figure 4f, *p* = 0.2710), HVLT (Figure 4g, *p* = 0.9404) or BAI (Figure 4h, *p* = 0.9054) as evaluated by permutation tests (*N* = 1,000 replicates). These results show that oculomotor parameters are able to account for a small to modest portion of the variance in cognitive scores in healthy individuals. Scores that show less individual variation in our sample population (MoCA) or assess anxiety as opposed to cognition (BAI) were the least well-explained by oculomotor models.

Additionally, the ability of PLS regression to predict clinical measures when including age in addition to oculomotor parameters as a model feature was evaluated (Supplementary Figure 1). When this demographic variable was included, the amount of variance explained by each model stayed the same or increased slightly (0–17% increase). The greatest improvement in model performance was observed for SDMT (Supplementary Figure 1d; *R*^2^ = 0.47, Adjusted *R*^2^ = 0.45) with an additional 17% in variance explained, followed by 7% for HVLT (Supplementary Figure 1f; *R*^2^ = 0.25, Adjusted *R*^2^ = 0.23) and 6% for TMTB (Supplementary Figure 1b; *R*^2^ = 0.26, Adjusted *R*^2^ = 0.23). The remaining models either had a small (1–2%) increase in variance explained (TMTA – Supplementary Figure 1a: *R*^2^ = 0.24, Adjusted *R*^2^ = 0.21; BAI – Supplementary Figure 1g: *R*^2^ = 0.15, Adjusted *R*^2^ = 0.11) or no change in performance (COWAT – Supplementary Figure 1c: *R*^2^ = 0.26, Adjusted *R*^2^ = 0.21; MoCA – Supplementary Figure 1e: *R*^2^ = 0.15, Adjusted *R*^2^ = 0.12). These findings indicate that for some cognitive scores, particularly those most correlated with age (SDMT, HVLT; Figure 1), including age in the model helps capture variance in cognitive performance not explained by oculomotor parameters alone. However, that oculomotor parameters explained a substantial amount of the variance in cognitive scores prior to including age suggests that these parameters capture meaningful individual differences in cognition beyond age-related effects.

**Figure 5:**
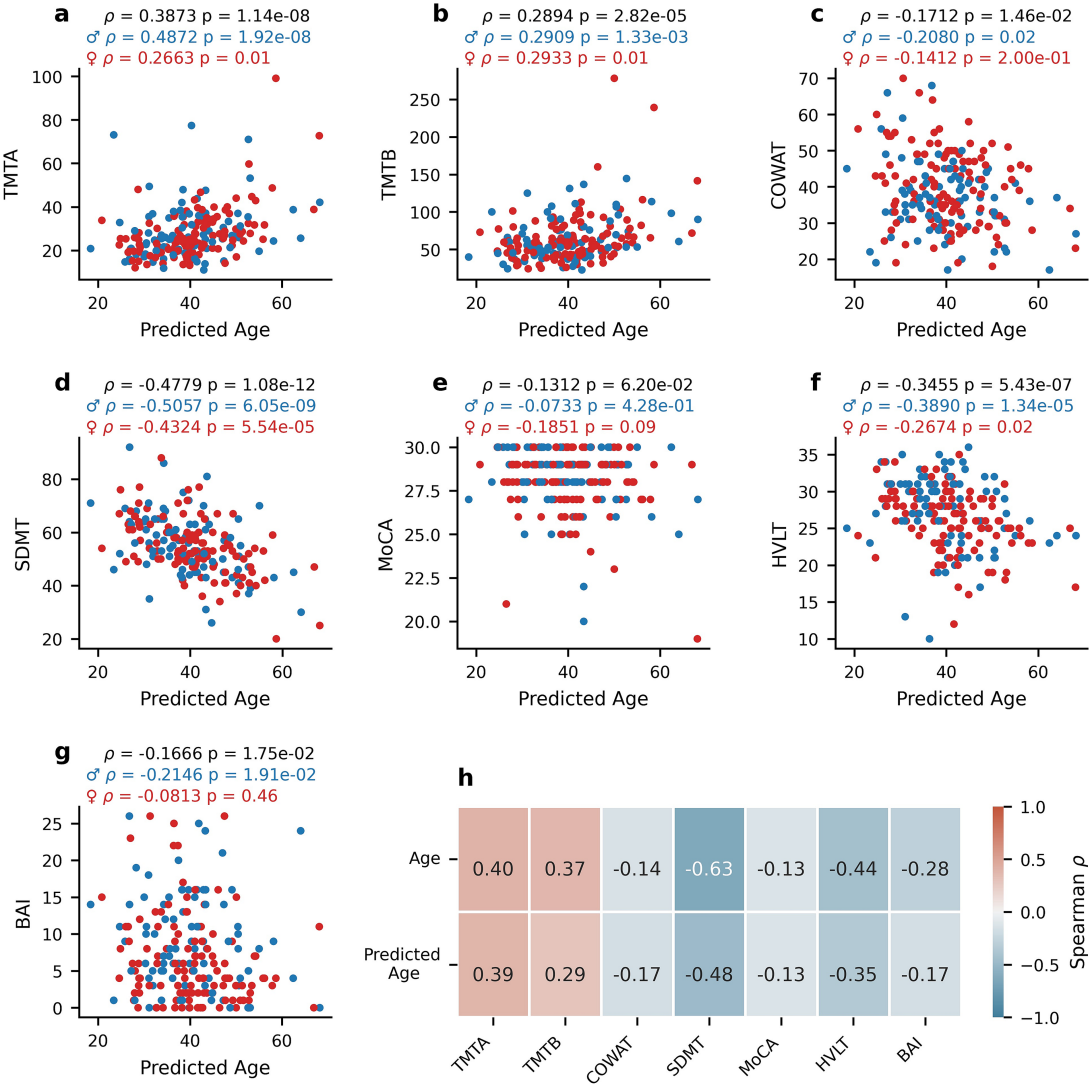
Relationship between oculomotor-predicted age and clinical measures. Scatterplots of the relationship between the oculomotor-based PLS predictions of age (predicted age) and clinical scores for healthy participants: TMTA **(a)**, TMTB **(b)**, COWAT **(c)**, SDMT **(d)**, MoCA **(e)**, HVLT **(f)**, and BAI **(g)**. Spearman’s rho correlation coefficients between true age or predicted age and each score **(h)**. Blue dots represent male participants and red dots represent female participants. Spearman’s rho and corresponding p-values are reported for the entire healthy participant dataset (black), for females only (red) and males only (blue) above panels **(a–g)**.

To explore whether oculomotor-predicted age captures meaningful variation related to cognitive function, age estimates from our PLS regression model (Figure 4a) and true chronological age were compared. Figure 5 depicts the relationship between predicted age and TMTA (Figure 5a, Spearman’s rho = 0.3873, *p* = 1.14*10^−8^), TMTB (Figure 5b, Spearman’s rho = 0.2894, *p* = 2.82*10^−5^), COWAT (Figure 5c, Spearman’s rho = −0.1712, *p* = 0.0146), SDMT (Figure 5d, Spearman’s rho = −0.4779, *p* = 1.08*10^−12^), MoCA (Figure 5e, Spearman’s rho = −0.1312, *p* = 0.0620), HVLT (Figure 5f, Spearman’s rho = −0.3455, *p* = 5.43*10^−7^), and BAI (Figure 5g, Spearman’s rho = −0.1666 *p* = 0.0175). Similar to the relationships with true age, the correlations between predicted age and TMTA (*p* = 0.0680), TMTB (*p* = 0.9870), COWAT (*p* = 0.6750), SDMT (*p* = 0.5320), MoCA (*p* = 0.4230), HVLT (*p* = 0.3080) and BAI (*p* = 0.3580) did not display sex differences as determined by permutation tests (*N* = 1,000 replicates). The correlations between predicted age and cognitive scores were also similar to those observed with true age in terms of magnitude of correlation and direction of the relationship. To confirm this, permutation tests (*N* = 1,000 replicates) were performed to assess whether the correlations between true age and clinical scores were statistically different from those between predicted age and clinical scores (Spearman’s rho correlations reported in Figure 5h). Apart from SDMT (*p* = 0.0420), correlations were not found to be significantly different: TMTA (*p* = 0.860), TMTB (*p* = 0.3750), COWAT (*p* = 0.7950), MoCA (*p* = 0.9740), HVLT (*p* = 0.2780), BAI (*p* = 0.2210). These findings indicate that oculomotor-predicted age varies with cognition in a manner comparable to chronological age, supporting its potential as a proxy for age-related cognitive change.

**Figure 6:**
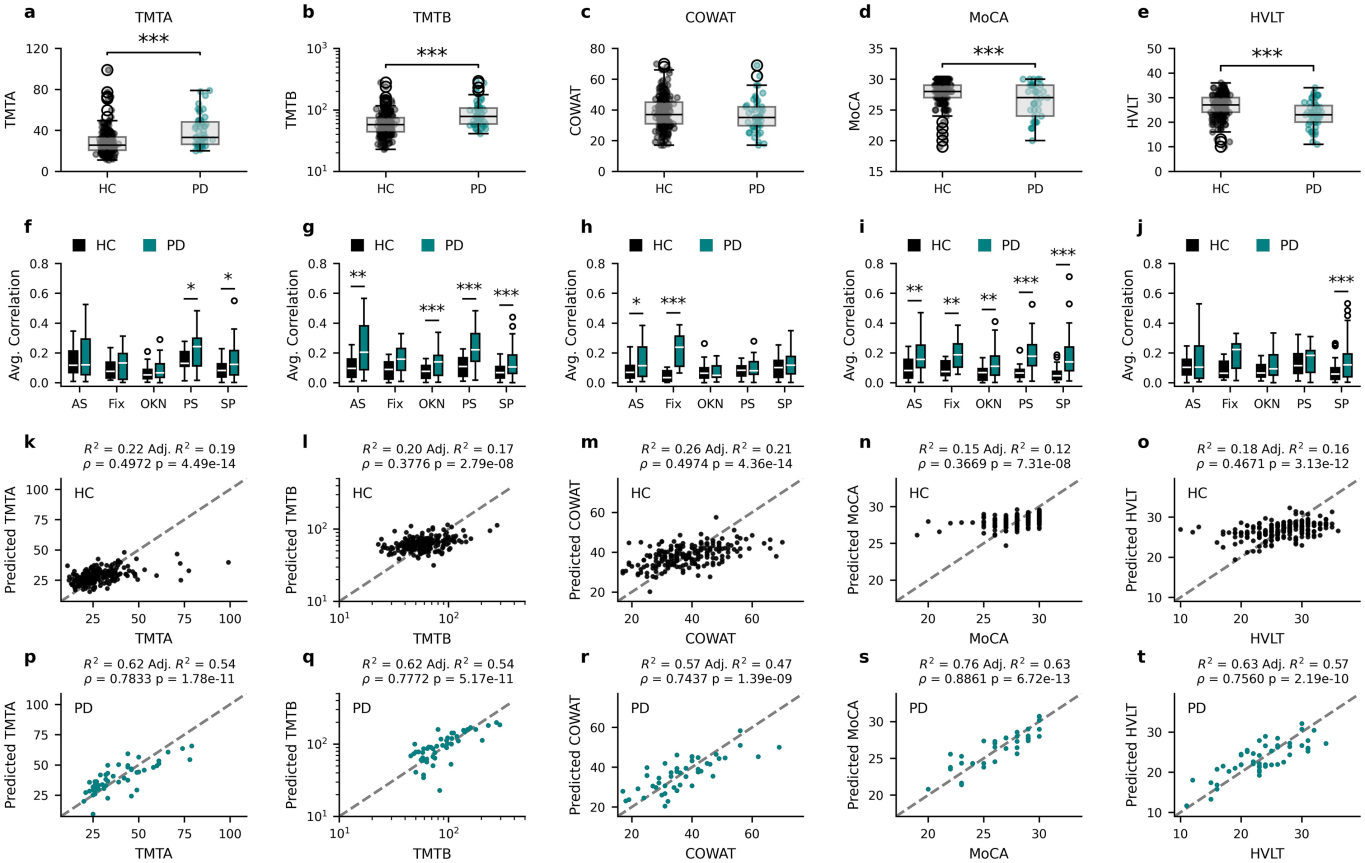
Oculomotor parameters are more predictive of cognitive measures for PD than HC participants. Cognitive score distributions for the healthy participants (Healthy Controls – HC) and PD patients from Koch et al. (24) for TMTA **(a)**, TMTB **(b)**, COWAT **(c)**, MoCA **(d)** and HVLT **(e)**. The corresponding average absolute correlation coefficient for each oculomotor task with each of the cognitive score distributions for HC and PD **(f–j)**. Relationships between the participants’ cognitive scores and the corresponding predicted value obtained by PLS regression analysis for HC **(k–o)** and PD **(p–t)**. AS, anti-saccade; Fix, fixation; OKN, optokinetic nystagmus; PS, pro-saccade; SP, smooth pursuit. * *p* < 0.05, ** *p* ≤ 0.01, *** *p* ≤ 0.001 (Mann–Whitney U corrected *p*-values).

Finally, to directly compare the ability of PLS regression models to predict cognitive scores from oculomotor parameters in healthy or PD participants, the PD data published in Koch et al. (24) was reanalyzed, the results of which are shown in Figure 6. The clinical outcome measures collected in both the neurologically intact individuals (Healthy Controls; HC) and PD patients from Koch et al. (24) are first reported, with TMTA (Figure 6a; Mann–Whitney U (MWU) = 2583.5, *p* = 7.683*10^−8^), TMTB (Figure 6b; MWU = 2651.0, *p* = 3.967*10^−7^), MoCA (Figure 6d; MWU = 4913.5, *p* = 0.0008) and HVLT (Figure 6e; MWU = 6857.5, *p* = 4.7113*10^−5^), but not COWAT (Figure 6c; MWU = 5416.5, *p* = 0.2288) scores differing significantly between the populations.

To assess whether the relationship between clinical outcome measures and oculomotor parameters is comparable in the neurologically intact and PD populations, Spearman’s rho correlation coefficients between cognitive scores and all oculomotor parameters within each population (as in Figure 2) were calculated and the average absolute correlation coefficients within each oculomotor task (anti-saccade (AS), fixation (Fix), optokinetic nystagmus (OKN), pro-saccade (PS) and smooth pursuit (SP)) were compared (Figures 6f–j). In general, the average correlations between cognitive scores and oculomotor parameters in PD patients had greater magnitude than those of HC participants, although this varied by score and task. To investigate this further, two-tailed Mann Whitney U tests were performed and corrected for multiple comparisons using the Benjamini-Hochberg procedure. All scores contained at least one task for which PD correlations were found to be statistically larger than HC correlations (Figures 6f–j). For full statistical results see Supplementary Table 3.

To further examine the stronger relationships between oculomotor parameters and cognitive scores in PD patients, new PLS regression models were fit for the PD group using the same procedures as for the neurologically intact population (see Methods). In comparison to Koch et al. (24), the current methods explore a greater number of oculomotor parameters and include PPCA imputation, which better leverages the full dataset of both participants and parameters. As such, the use of the same method enabled comparison of model performance between the HC and PD groups. The relationship between HC model predictions and the clinical outcome measures common to both populations are reproduced from Figure 4 in Figures 6k–o to aid in the comparison with the same relationships in the PD population in Figures 6p–t.

Notably, the oculomotor parameter-based PLS models explain a greater proportion of cognitive score variance in PD patients (47–63%) than in neurologically intact individuals (12–28%), and predicted scores are more closely related to true scores (Spearman’s rho PD: 0.74–0.89 vs. HC: 0.37–0.50). Specifically, the PLS regression model for TMTA explains 54% of PD TMTA scores (Figure 6o; *R*^2^ = 0.62, Adjusted *R*^2^ = 0.54, Spearman’s rho = 0.7833, *p* = 1.78*10^−11^) in comparison to the 19% variance (rho = 0.4972, *p* = 4.49*10^−14^) explained by the equivalent model in HCs. The TMTB PLS regression model explains 54% of PD patient TMTB scores (Figure 6q; *R*^2^ = 0.62, Adjusted *R*^2^ = 0.54, Spearman’s rho = 0.7772, *p* = 5.17*10^−11^) compared to the 17% variance (rho = 0.3776, *p* = 2.79*10^−8^) explained by the equivalent model in HCs. Similarly, the PLS regression model for the PD population explains 47% of PD COWAT scores (Figure 6r; *R*^2^ = 0.57, Adjusted *R*^2^ = 0.47, Spearman’s rho = 0.7437, *p* = 1.39*10^−09^) instead of the 21% variance (rho = 0.4974, *p* = 4.36*10^−14^) explained by the model for the HC population. For MoCA scores, the PLS regression model for PD patients explains a much larger percentage (63%) of the PD population’s MoCA scores (Figure 6s; *R*^2^ = 0.76, Adjusted *R*^2^ = 0.63, Spearman’s rho = 0.8861, *p* = 6.72*10^−13^) compared to the 12% explained variance (rho = 0.3669, *p* = 7.31*10^−8^) of the PLS regression for the HC population. Finally, the PLS regression model for HVLT for PD patients also explains more variance (57%) in HVLT scores (Figure 6t; *R*^2^ = 0.63, Adjusted *R*^2^ = 0.57, Spearman’s rho = 0.7560, *p* = 2.12*10^−7^) than the model for HCs (16% variance explained, rho = 0.4671, *p* = 2.19*10^−10^). Additionally, permutation tests (*N* = 1,000 replicates) were carried out to assess whether the relationship between predicted and true scores (as quantified by the Spearman’s rho values) differed between the groups for each score. The PLS-predicted values were significantly more correlated with the true values for the PD models than HC models for all scores: TMTA (*p* = 0.0129), TMTB (*p* = 0.006), COWAT (*p* = 0.036), MoCA (*p* = 0.003), HVLT (*p* = 0.015). Taken together, the PLS regression models leverage information present in the oculomotor parameters to explain more of the variance in clinical outcome measures in PD patients than in neurologically intact individuals.

## Discussion

This study’s primary objective was to determine the extent to which cognitive ability can be estimated via oculomotor parameters in a large sample of neurologically intact individuals. To measure such parameters, a novel mobile eye-tracking software was used that functions using the standard camera of an iPad Pro, and has been used previously to examine similar relationships in PD (24) and MS patients (25). To evaluate cognitive ability, four of the cognitive domains outlined in the Movement Disorder Society Task Force Guidelines (29) were measured for comparisons with previous findings in PD participants: MoCA, COWAT, HVLT and TMTA/B. The Symbol Digit Modalities Test (SDMT) was additionally included to assess cognitive processing speed and the Beck Anxiety Inventory (BAI) was also added to assess participant anxiety levels as part of the clinical assessment.

### Relationship between oculomotor parameters and cognitive domains

To determine the extent to which oculomotor parameters could be used to estimate clinical measures of cognition in neurologically intact individuals, partial least squares (PLS) regression, which accounts for multicollinearity among the predictor variables (oculomotor parameters), was used. This approach yielded adjusted *R*^2^ values (Figures 4b–g) between 0.10 (BAI) and 0.28 (SDMT) for predictions of cognitive scores. Additionally, a previously reported (24) PD dataset was re-analyzed for a direct comparison with the healthy participants. Notably, larger proportions of the variance in clinical outcome measures could be explained by the oculomotor parameters of the PD individuals than from those of the healthy group (Figures 6k–t), which was also reflected in greater correlations between the actual and predicted clinical measures in the PD dataset, ranging from an increase of 0.25 for COWAT to 0.52 for MoCA.

Figures 6f–j depicts the average absolute correlation coefficients between a given clinical measure and the oculomotor parameters measured from a specific eye-tracking task (i.e., anti-saccade, fixation, pro-saccade, smooth pursuit, and optokinetic nystagmus) for HC and PD. Notably, the mean correlation coefficients associated with anti-saccade, pro-saccade, and smooth pursuit parameters were systematically higher in the PD cohort relative to the neurologically intact group. This differential likely underlies the reduced explanatory power of the oculomotor-based models for clinical cognitive scores observed in the healthy control sample. These findings are in line with those from the previous report on PD patients (24), in which anti-saccade and pro-saccade parameters contributed the most to the clinical outcome measure predictive models. Indeed, there is ample evidence that anti-saccade tasks, in particular, tap into several executive and frontal cognitive processes, including psychomotor speed, visual search, attention task-switching, and inhibition (49–52) that are affected by PD. Similarly, several pro-saccade characteristics such as latency, duration, peak, and average velocity predict decline in global cognition, executive function, attention, and memory in PD patients (18, 53). Of note, the score that was predicted most poorly for the HC group was MoCA, which is an assessment of general cognitive ability that does not show large variation in healthy individuals. It is therefore not surprising that the variation in this score was more easily captured by oculomotor parameters in PD patients.

Taken together, these findings suggest that in the absence of significant impairment of brain function, as typically observed in neurological disorders, the oculomotor system may offer a more subtle signal with respect to predicting cognitive ability. This underscores the strength of oculomotor analysis as a tool that is sensitive to deviations from typical brain function and provides a baseline of the relationship between eye movements and cognitive scores in neurologically intact individuals without the underlying confound of neuropathology. Our results indicate that it is therefore likely that the link between eye-tracking data and cognition is mediated by the functional integrity of brain circuits, which highlights the utility of oculomotor analysis for the detection of neurocognitive disorders.

Although the explanatory power of the PLS regression models in neurologically intact individuals was modest, these results remain scientifically informative. In complex, multifactorial systems such as cognition, modest *R*^2^ values are expected because performance reflects the joint influence of diverse neural, experiential, and environmental factors. The fact that oculomotor parameters nevertheless accounted for a significant portion of variance suggests that they capture mechanistically relevant aspects of brain function. In particular, parameters derived from pro-saccade and anti-saccade tasks contributed most strongly to the latent components, consistent with the involvement of fronto-striatal and parietal networks known to support inhibitory control, attentional shifting, and processing speed (54–56). This indicates that even subtle variation in oculomotor control among healthy individuals reflects differences in the efficiency of higher-order cognitive systems. By contrast, the substantially greater variance explained in the PD cohort underscores how neurodegeneration amplifies the coupling between oculomotor and cognitive processes. Thus, while the analyses in the present context were not designed to provide clinical predictions at the individual level, they nevertheless provide mechanistic insight into shared neural substrates across motor and cognitive domains, and highlight the potential of oculomotor markers as sensitive indicators of early or subclinical changes in brain function.

The expected impact of oculomotor functions also varies across the clinical measures. For tasks that explicitly depend on visual scanning and rapid saccadic movements, such as the Trail Making Tests (TMTA/B) and the Symbol Digit Modalities Test (SDMT), oculomotor efficiency likely contributes directly to performance. In contrast, tests such as the Hopkins Verbal Learning Test (HVLT) and Controlled Oral Word Association Test (COWAT) primarily index memory and verbal fluency, and any oculomotor contribution is likely indirect, reflecting shared reliance on fronto-striatal and parietal circuits that support executive control and attention. The MoCA, as a composite measure including visuospatial and executive components, likely reflects a mixture of both direct and indirect influences. Finally, the BAI is least tied to oculomotor control, with effects expected to be indirect at best. Based on this rationale, the anticipated influence of oculomotor functions can be ranked as strongest for SDMT and TMTA/B, intermediate for MoCA, HVLT, and COWAT, and weakest for BAI. This framework highlights how eye-movement metrics can serve as both direct measures of visual-motor efficiency and indirect markers of broader neural integrity underlying cognition.

### Effect of sex on the relationship between oculomotor parameters and cognition

Although the examination of potential sex differences in eye-tracking and oculomotor research has, to a large extent, been largely ignored or underreported, studies that have directly investigated this issue have reported mixed results. For instance, Coors et al. (57) noted that only a few of their investigated oculomotor parameters exhibited weak sex differences and both Mathew et al. (58) and Takahashi et al. (59) failed to demonstrate reliable sex differences. In contrast, studies with very large sample sizes [Bargary et al. (60) – *N* > 1,000 participants; Carrick et al. (61) – *N* > 23,000 participants] have reported sex differences across a range of oculomotor parameters. The requirement of such large sample sizes to demonstrate inter-sex differences raises the question of the clinical or biological relevance of the magnitude of these sex differences.

In this study, potential sex differences in the manner in which oculomotor parameters relate to cognition were investigated and no differences in the PLS model *R*^2^ values between males and females (see Figure 4) were found. Furthermore, the relationship between age and clinical scores was compared between males and females (see Figure 2), and no differences in the sex-based Spearman’s rho values were found.

### Relationship between age, cognition, and oculomotor parameters

There is ample evidence in the literature supporting the effect that age has on cognitive ability, as measured via neuropsychological tests, consistent with our present findings and highlighted in Figure 1a. For instance, the ability to successfully complete TMTA and TMTB tends to slowly decline until the age of 60, at which point the slope of the decline increases (62). This decline is in line with the positive correlations reported here as worsening is evidenced by the increased time required to complete the tasks. Both SDMT (63) and HVLT (64) scores are also known to strongly correlate with age. It is also well established that several eye-movement parameters vary as a function of age, consistent with our findings that many oculomotor parameters significantly correlate with age (Figure 2). For instance, saccade latency and error rate in the antisaccade task increase with age (30, 32, 65), whereas fixation stability tends to decrease and the frequency of catch-up saccades during smooth pursuit increases with age (66).

The purpose here, however, was to move beyond the known relationships between age and specific parameters, and instead to attempt to predict an individual’s age based on their measured oculomotor parameters. The idea is akin to recent studies attempting to develop biomarker-based age models to infer an individual’s biological or “brain” age using MRI (67, 68), and where the difference between one’s estimated age and chronological age, the “brain age gap” (BAG), has been proposed as a marker of brain health or brain functional integrity. We suspect that testing the oculomotor-based age model on a clinical population would produce a larger BAG than in a group of healthy controls – unfortunately, this could not be specifically tested in the current study due to the critical absence of a withheld age-matched control group.

Nonetheless, the oculomotor-based age model performed reasonably as a first attempt, explaining roughly 33% of the age variance in the subject sample (see Figure 4a). The accuracy of the age predictions was further examined by investigating their relationships with the clinical test scores. As seen in Figure 5, the “predicted age” correlation coefficients were roughly on par with the coefficients obtained using the participant’s true age, providing an additional line of evidence linking oculomotor parameters and cognition. Next steps in this specific line of research include future studies pairing known markers of biological age with eye tracking to develop a more robust eye-tracking model of biological age or brain age, which in turn could lead to the development of a low-cost, non-invasive, and scalable indicator of brain health and disease.

To assess whether anxiety might confound the observed relationships between oculomotor features and cognitive outcome measures, we included the BAI score in our analyses. The BAI was not significantly correlated with any oculomotor parameters (Figure 2), and PLS regression models in the HC group demonstrated the worst predictive power for anxiety in terms of variance explained (Figure 4). Therefore, we do not believe that anxiety levels played a role in driving the observed results.

### Study limitations

A first limitation relates to the extent to which participants were truly cognitively intact, given that they self-reported as such. Using the cutoff score of 23 outlined in the updated MoCA criteria by Carson et al. (69), half a dozen of the participants would in fact be classified as having mild cognitive impairment. However, given that these participants represent less than 3% of our total study sample, it is unlikely that they significantly biased any of the reported findings. Moreover, their inclusion likely makes the dataset more generalizable to the general population without diagnosed neurological disorders.

Although cognitive ability is inferred here via variance explained by oculomotor parameters, partial least squares regression analyses produce inference models, which do not necessarily guarantee strong predictive abilities. To claim that disease, cognitive ability, or even age, in a single individual can be estimated, predictive models would need to be validated with an independent dataset. Although several cognitive domains were investigated, not all of them were sampled, and stronger relationships with oculomotor parameters might exist with other cognitive functions captured with different neuropsychological tests not included in the present study. In particular, the cognitive domains sampled were limited to those typically associated with decline in PD.

The sample sizes of our two comparison groups were unequal, and perhaps more importantly, were not age-matched. However, the age distributions of our groups were roughly representative of their associated population distributions (adult neurologically intact individuals and individuals with mild-to-moderate PD), suggesting that our findings are likely generalizable to those population sets. Finally, another minor limitation relates to data loss caused by the camera position relative to the iPad Pro tablet (at the top), which makes it difficult to accurately detect the eyes when looking at the bottom of the screen for some subjects due to the eyelids partially obscuring relevant gaze estimation markers – see Supplementary methods for further details. As a result, data collected from large amplitude downward saccades were removed from all subjects and all analyses.

Another limitation relates to the assumption of symmetrical velocity profiles during saccades. Neurological conditions such as Parkinson’s disease often produce asymmetric velocity profiles, with an extended deceleration phase relative to acceleration. This asymmetry may introduce measurement error in estimates of latency and peak velocity. If present, such errors could lead to the underestimation of the strength of observed associations between eye movement parameters and clinical outcomes. Future iterations of the model will explore relaxing the symmetry constraint to better accommodate atypical velocity profiles observed in these populations.

Finally, one additional methodological consideration concerns the use of Spearman’s rho. While this statistic is less sensitive to skew and outliers than Pearson’s correlation, it assumes iid observations and is affected by ties in the data. Given our study design, where each participant contributed one observation, independence was satisfied. Although ties occurred for some discrete clinical measures (e.g., MoCA, SDMT), their influence was minimized by the average-rank procedure. Thus, while Spearman’s rho captures monotonic rather than strictly linear associations, we believe it provided the most appropriate framework for relating oculomotor and cognitive variables in this dataset.

## Conclusion

The present study set out to investigate the relationship between oculomotor parameters and clinical scores of cognition, age, and sex. Within neurologically intact individuals, no significant differences between males and females regarding the relationship between the examined clinical scores and oculomotor parameters were observed. Overall, the findings demonstrate that while oculomotor parameters show a modest but statistically reliable relationship with cognitive performance in neurologically intact individuals, their predictive value is substantially greater in individuals with a neurodegenerative disorder (PD). This pattern suggests that the link between cognitive function and eye-tracking parameters may be amplified in the context of neurodegeneration, likely due to disruptions in shared neural systems supporting both motor control and cognition. In healthy individuals, the relationship appears more subtle, consistent with the idea that the oculomotor system reflects broader aspects of brain function that only become more diagnostically informative when those systems are compromised. Finally, our results reveal a moderate association between age and several oculomotor parameters, suggesting that while oculomotor analysis alone may not precisely estimate an individual’s “brain age” or biological age, it could nonetheless contribute meaningfully to future predictive models when combined with other relevant biomarkers.

## Data Availability

The raw data supporting the conclusions of this article will be made available by the authors, without undue reservation.

## Supplementary material

SUPPLEMENTARY FIGURE 1 Predictions of clinical measures from oculomotor parameters using PLS regression models with the addition of age as a predictor. Scatterplots of healthy participants’ clinical scores compared to the corresponding predicted value obtained by PLS regression analysis using oculomotor parameters and age as predictors: TMTA **(a)**, TMTB **(b)**, COWAT **(c)**, SDMT **(d)**, MoCA **(e)**, HVLT **(f)**, and BAI **(g)**. Blue dots represent male participants and red dots represent female participants. *R*^2^ and adjusted *R*^2^ values are reported for the entire healthy dataset (black), as well as *R*^2^ values for females only (red), and males only (blue) above each panel. No significant differences in model performance between males and females were found when tested via permutation tests (*N* = 1000 replicates): TMTA (*p* = 0.4702), TMTB (*p* = 0.9604), COWAT (*p* = 0.8408), SDMT (*p* = 0.9938), MoCA (*p* = 0.2792), HVLT (*p* = 0.6940), BAI (*p* = 0.6534).
