# Supplementary material for: Oculomotor indicators of cognitive performance are modulated by neurodegeneration
Source: Front Neurol. 2025 Nov 10;16:1649745. doi: 10.3389/fneur.2025.1649745 (PMC12640912; doi:10.3389/fneur.2025.1649745)
Supplement: SUPPLEMENTARY FIGURE 1 — Predictions of clinical measures from oculomotor parameters using PLS regression models with the addition of age as a predictor. Scatterplots of healthy participants’ clinical scores compared to the corresponding predicted value obtained by PLS regression analysis using oculomotor parameters and age as predictors: TMTA (a), TMTB (b), COWAT (c), SDMT (d), MoCA (e), HVLT (f), and BAI (g). Blue dots represent male participants and red dots represent female participants. R2 and adjusted R2 values are reported for the entire healthy dataset (black), as well as R2 values for females only (red), and males only (blue) above each panel. No significant differences in model performance between males and females were found when tested via permutation tests (N = 1000 replicates): TMTA (p = 0.4702), TMTB (p = 0.9604), COWAT (p = 0.8408), SDMT (p = 0.9938), MoCA (p = 0.2792), HVLT (p = 0.6940), BAI (p = 0.6534). [file Data_Sheet_1.pdf]

Supplementary Materials

Methods

Oculomotor Tasks

Fixation task: Participants had to fixate a stationary target for 7 s, presented sequentially at five different locations (one central and 4 eccentric locations). The eccentric positions were located 10 degrees of visual angle left and right from the center and 14 degrees of visual angle up and down from the center.

Pro-saccade task: Participants had to initially fixate a central fixation cross, which disappeared after a random period of 1.0–3.5 s, after which a different target reappeared elsewhere on the screen at an eccentric location for 1.5 s either to the left or right, above, or below the central fixation point. Participants were instructed to move their gaze as quickly as possible to the new target location. Both Small (5° horizontal, 6o vertical) and Large (10° horizontal, 12° vertical) amplitude eccentric target distances were used. Each target location was sampled 3 times, for a total of 24 trials.

Anti-saccade task: Participants had to initially fixate a central fixation cross, which disappeared after a random period of 1.0–3.5 s, after which a different target reappeared elsewhere on the screen at an eccentric location (10°) either to the left or right of the center. Participants were instructed to move their gaze as quickly as possible in the opposite direction to the new target location. After being displayed for only 100 ms, the target disappeared, and the screen was left blank for a predetermined duration of time. Following the blank screen, a symbol appeared in the opposite location of where the initial stimulus appeared (i.e., where the participant should be looking). This symbol consisted of a white square with an arrow inside oriented in one of 4 random directions: either left, right, up, or down. The addition of the symbol detection portion of

the task was inspired by a previous study (Guitton et al., 1985), in which correct identification of a second visual symbol was contingent on successful execution of the anti-saccade. In our adaptation, this allowed for an additional, behaviorally anchored measure of anti-saccade accuracy, offering greater granularity in evaluating task performance.

Three different difficulty levels were used, each comprising 8 trials. In the Slow trials, the blank screen period lasted 1,200 ms and the arrow symbol duration of 400 ms, in the Medium trials the blank screen period lasted 800 ms and the arrow symbol duration of 250 ms, and in the Fast trials the blank screen period lasted 550 ms and the arrow symbol duration of 100 ms. After each trial, a screen was displayed for 5 s, prompting the user to answer which symbol they saw by directing their gaze towards the arrow orientation corresponding to what they believed was the correct answer. The adjustment of these specific task parameters across levels was intended to specifically increase the difficulty associated with correctly identifying the symbol presented at the end of each anti-saccade trial.

Smooth pursuit task: Participants here were first required to fixate a central fixation cross of variable duration (1–2 s). Once the fixation cross disappeared, a moving target (that could either go up, down, left or right) appeared on screen, for which the participants were instructed to follow with their gaze. Step–ramp paradigm of smooth pursuit at constant velocity was used, whereby the initial position of the moving target was positioned offset from the central fixation point, on the opposite side of the motion direction (Supp. Figure 1d). For instance, in a trial of rightward smooth pursuit, the motion target would first appear to the left of the central fixation point (i.e., the step) and then move in the opposite direction (rightward) at a constant velocity (i.e., the ramp). The trial terminated when the target reached the 10° position, either left, right, above, or below center. A total of twelve trials were performed, three in each direction, with a target that moved at one of three constant velocities (Slow: 8.65°/s, with a step size of 1.5°) (Medium: 17.1°/s, with a step size of 2.7°) (Fast: 25.9°/s, with a step size of 4.1°).

Optokinetic nystagmus task: Participants were first required to fixate a central cross for three seconds, after which a 100% contrast horizontally moving vertical square wave grating would appear on screen. The grating stimulus was presented full screen with a fundamental spatial frequency of one cycle per 2.5 degrees. Four different direction and velocity combinations were presented to each participant, each preceded by the fixation cross and each last for 15 s: 1) leftward motion at 4 degrees per second, 2) rightward motion at 4 degrees per second, 3) leftward motion at 8 degrees per second, and 4) rightward motion at 8 degrees per second.

Oculomotor parameter extraction

Eye movement parameter extraction was performed offline using ETNA™'s automatic proprietary analysis pipeline. Before parameter extraction, all gaze signals were processed and non-saccadic artifacts (e.g., blinks) were removed by the software's analysis pipeline. Gaze signals were also denoised using Savitzky-Golay filters. An adaptive, velocity-based algorithm was used for saccade detection, based on the work of (Schweitzer & Rolfs, 2020). Saccade parameters were then obtained by fitting a parametric model for saccadic waveforms (Dai et al., 2016), which reproduces the established relationship between peak saccadic angular velocity and saccadic amplitude (i.e., the saccadic main sequence) to the the gaze position signals acquired during the pro-saccade and anti-saccade tasks. This model fitting provides saccade parameters such as the saccade latency, amplitude and peak velocity. Although based on the main sequence, the parametric model does not assume a fixed main sequence profile. Rather, it fits saccadic waveforms by estimating parameters directly from the gaze position signal. This allows for flexible modeling of saccades that deviate from normative patterns, including those commonly observed in neurodegenerative conditions.

Fixation parameters were averaged across all positions, resulting in a single set of fixation parameters. Pro-saccades parameters were averaged across locations and dimensions, with

the exception of the large amplitude downward saccades, which were removed from all analyses. This removal was done because data for these trials was often absent due to difficulty in detecting the eyes caused by the lowering of the eyelids during large downward saccades. This yielded two sets of prosaccade parameters: one for large amplitude saccades (excluding the data from the downward saccade trials) and one for small amplitude saccades. Anti-saccades parameters were averaged across directions (i.e. left and right), resulting in three sets of anti-saccade parameters, one per difficulty level. Smooth pursuit parameters were averaged across target directions for a given target velocity, resulting in three different parameter sets, one for each velocity used. Finally, OKN parameters were averaged across directions, resulting in one parameter set per velocity used. The extracted oculomotor parameters included but were not limited to: saccadic intrusion frequency and amplitude (fixation), saccadic latency, velocity and precision (pro-saccades), direction-specific latency and directional success rate (anti-saccades), amplitude of drift and velocity of return saccade (optokinetic nystagmus), pursuit velocity and the quantification (e.g. count and amplitude) of catch-up saccades (smooth pursuit).

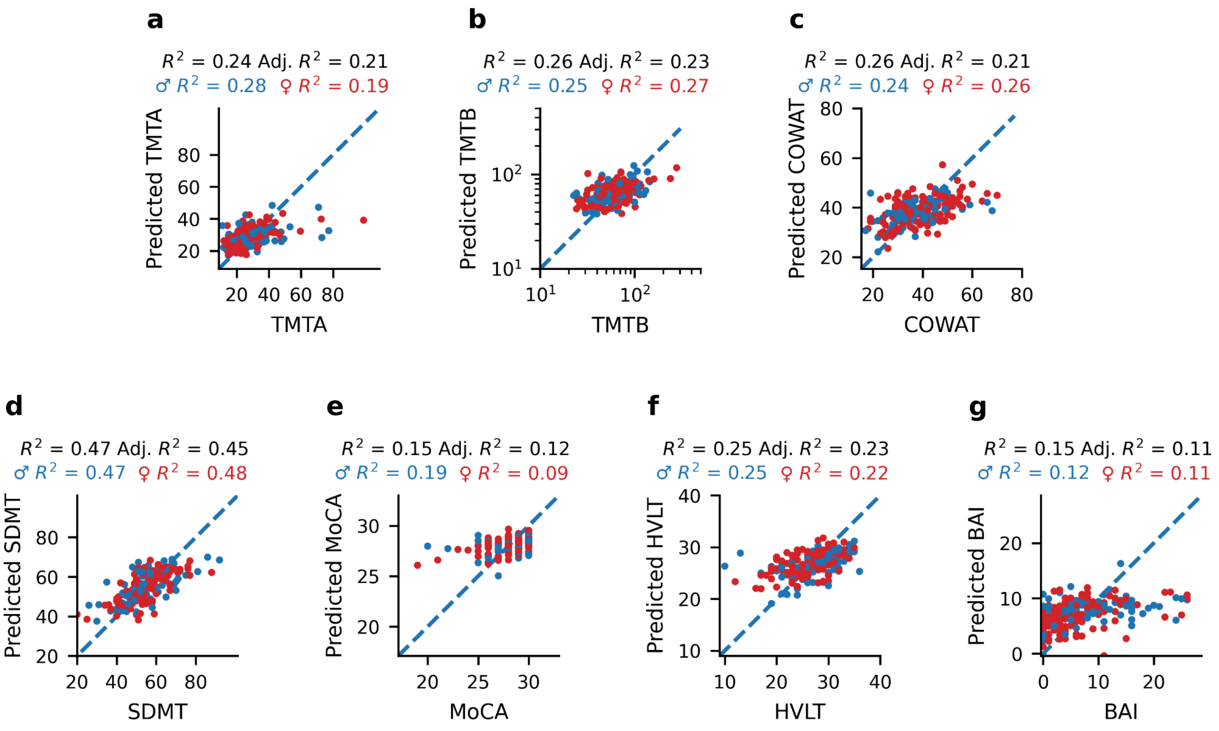

Supplementary Figure 1: Predictions of clinical measures from oculomotor parameters using PLS regression models with the addition of age as a predictor. *Scatterplots of healthy participants' clinical scores compared to the corresponding predicted value obtained by PLS regression analysis using oculomotor parameters and age as predictors: TMTA (a), TMTB (b), COWAT (c), SDMT (d), MoCA (e), HVLT (f), and BAI (g). Blue dots represent male participants and red dots represent female participants. R^2 and adjusted R^2 values are reported for the entire healthy dataset (black), as well as R^2 values for females only (red), and males only (blue) above each panel. No significant differences in model performance between males and females were found when tested via permutation tests (N=1000 replicates): TMTA (p=0.4702), TMTB (p=0.9604), COWAT (p=0.8408), SDMT (p=0.9938), MoCA (p=0.2792), HVLT (p=0.6940), BAI (p=0.6534).*
